# Survival After Implementation of a Decision Support Tool to Facilitate Evidence-Based Cancer Treatment

**DOI:** 10.1200/CCI.23.00001

**Published:** 2023-06-21

**Authors:** Rohit P. Ojha, Yan Lu, Kalyani Narra, Rachel J. Meadows, Aaron W. Gehr, Emmanuel Mantilla, Bassam Ghabach

**Affiliations:** ^1^ Center for Epidemiology & Healthcare Delivery Research, JPS Health Network, Fort Worth, TX; ^2^ Oncology and Infusion Center, JPS Health Network, Fort Worth, TX; ^3^ Acclaim Neurology, JPS Health Network, Fort Worth, TX

## Abstract

**PURPOSE:**

Decision support tools (DSTs) to facilitate evidence-based cancer treatment are increasingly common in care delivery organizations. Implementation of these tools may improve process outcomes, but little is known about effects on patient outcomes such as survival. We aimed to evaluate the effect of implementing a DST for cancer treatment on overall survival (OS) among patients with breast, colorectal, and lung cancer.

**METHODS:**

We used institutional cancer registry data to identify adults treated for first primary breast, colorectal, or lung cancer between December 2013 and December 2017. Our intervention of interest was implementation of a commercial DST for cancer treatment, and outcome of interest was OS. We emulated a single-arm trial with historical comparison and used a flexible parametric model to estimate standardized 3-year restricted mean survival time (RMST) difference and mortality risk ratio (RR) with 95% confidence limits (CLs).

**RESULTS:**

Our study population comprised 1,059 patients with cancer (323 breast, 318 colorectal, and 418 lung). Depending on cancer type, median age was 55-60 years, 45%-67% were racial/ethnic minorities, and 49%-69% were uninsured. DST implementation had little effect on survival at 3 years. The largest effect was observed among patients with lung cancer (RMST difference, 1.7 months; 95% CL, –0.26 to 3.7; mortality RR, 0.95; 95% CL, 0.88 to 1.0). Adherence with tool-based treatment recommendations was >70% before and >90% across cancers.

**CONCLUSION:**

Our results suggest that implementation of a DST for cancer treatment has nominal effect on OS, which may be partially attributable to high adherence with evidence-based treatment recommendations before tool implementation in our setting. Our results raise awareness that improved process outcomes may not translate to improved patient outcomes in some care delivery settings.

## Teaser

Implementation of a decision support tool for cancer treatment has nominal effect on survival.

## INTRODUCTION

Evidence-based treatment is intended to optimize clinical outcomes for patients with cancer, but the rapid evolution of evidence and complexity of cancer poses challenges for translating evidence to practice. Accessibility and ease of implementation are key facilitators of delivering evidence-based treatment.^1^ Commercial and locally developed decision support tools (DSTs) are available to facilitate evidence-based cancer treatment.^2-4^ These DSTs (sometimes referred to as clinical pathways software) are available as standalone software or integrated into electronic health record (EHR) systems, which can enhance accessibility and ease implementation. Consequently, DSTs are increasingly promoted and implemented in cancer care delivery organizations across the United States.^2-5^ Nevertheless, evidence about the effects of implementing DSTs for cancer treatment on patient outcomes is limited.

**CONTEXT**

**Key Objective**
We aimed to evaluate the effect of implementing a decision support tool (DST) for cancer treatment on overall survival among patients with breast, colorectal, and lung cancer.
**Knowledge Generated**
DST implementation had little effect on survival at 3 years. The largest effect was observed among patients with lung cancer (restricted mean survival time difference, 1.7 months, 95% confidence limit [CL], –0.26 to 3.7; mortality risk ratio, 0.95; 95% CL, 0.88 to 1.0). Adherence with tool-based treatment recommendations was >70% before and >90% across cancers.
**Relevance**
DSTs for cancer treatment arguably have benefits that may justify implementation such as improvements in process outcomes such as care standardization, increased use of evidence-based treatments, and efficiency. Our results may be useful for raising awareness that improved process outcomes may not translate to improved patient outcomes in some care delivery settings. Continuous evaluation of DSTs is needed to understand the context in which these tools can improve patient outcomes.

Recent systematic reviews identified studies of DST implementation and outcomes for patients with cancer.^6,7^ The cumulative evidence from these reviews and two additional studies^8,9^ published subsequently suggests that DST implementation generally improved process outcomes such as care standardization (ie, reduced treatment variation), increased use of evidence-based treatments, and efficiency. Nevertheless, improved process outcomes may not necessarily translate to improved clinical outcomes,^7,10^ particularly the patient-important outcome of survival.^11^ Some studies of DSTs assessed survival,^12-14^ but methodologic limitations of these studies preclude valid inference.^6,7,15^ In addition, these studies were limited to one cancer type. A systematic evaluation of the effect of DST implementation on survival may generate useful information about whether the benefits extend beyond process outcomes.^3,4,7^ Our cancer center implemented a DST in December 2015, which allowed opportunity for such evaluation. Therefore, we aimed to evaluate the effect of implementing a DST on overall survival (OS) among patients with breast, colorectal, and lung cancer.

## METHODS

### Study Design

We emulated a single-arm trial with historical comparison at a single institution to address the aim.^16,17^ This quasi-experimental framework was most relevant, given that the DST (ie, the intervention) was universally implemented in our setting.^18^ This study was approved by the North Texas Regional Institutional Review Board with waiver of informed consent (IRB# 2017-109).

### Study Population

The John Peter Smith (JPS) Oncology and Infusion Center is a Comprehensive Community Cancer Program in an urban safety-net health system and the primary source of care for socioeconomically marginalized populations in Tarrant County, Texas.^19,20^ We used data from the institutional cancer registry to identify our study population. This registry includes patient characteristics, cancer staging and tumor characteristics, first course treatment, and outcomes observed during follow-up for all patients age 18 years and older who were treated at JPS Oncology and Infusion Center since January 1, 2008. Certified cancer registrars manage data collection, which is required to meet Commission on Cancer standards.^21^ Eligible individuals were age 18 years and older; diagnosed with histologically confirmed first primary breast, colorectal, or lung cancer between December 2013 and December 2017; and received first-line treatment at JPS Oncology and Infusion Center. Breast, colorectal, and lung cancer are the most common cancer types in our population. Small sample sizes precluded the inclusion of other cancer types.

### Intervention

Our intervention of interest was implementation of a commercial DST (ClinicalPath [formerly Via Oncology], Elsevier).^22^ This DST was implemented at the JPS Oncology and Infusion Center in December 2015. The intervention group thus comprised eligible individuals diagnosed between January 2016 and December 2017 (ie, after the DST was implemented and inclusive of a 1-month washout period to avoid combined effects^23^). The historical comparison group comprised eligible individuals diagnosed between December 2013 and November 2015 (ie, usual care on the basis of provider discretion before the DST was implemented).

The DST was developed by oncologists and uses trial evidence and real-world evidence to generate standardized treatment recommendations and inform care decisions on the basis of tumor characteristics with sequential prioritization of efficacy, toxicity, and cost.^24^ Cost is only a consideration if efficacy and toxicity are comparable between two regimens.^24^ This tool can be used standalone, as currently in our setting, or integrated with an EHR system. The tool also includes embedded access to National Comprehensive Cancer Network guidelines if treatment alternatives are needed because of contraindications, patient refusal, etc. Although not integrated into our EHR system, the DST is accessible to oncologists through the EHR using an embedded link or through a standard web browser, which allows oncologists an alternate access point and opportunity to use the DST for generating treatment recommendations at their convenience. Adherence to treatment recommendations was encouraged through quality incentives (financial incentive for meeting quality metric of organizational treatment adherence >90%), peer comparison (quarterly report that summarizes the individual provider performance *v* organizational performance), and documentation reminders (daily system-generated and monthly internal calendar reminders). Oncologists document adherence with tool-based treatment recommendations within the decision support software. If an oncologist does not follow the recommendation, a senior oncologist must review and document confirmation that deviation from the recommendation was clinically justified.

### Outcome and Covariates

Our outcome of interest was 3-year OS, where vital status was ascertained through multiple sources (eg, Bureau of Vital Statistics, Social Security Death Index, Texas Obituaries, Fort Worth Star-Telegram Obituaries, Tarrant County Medical Examiner's Office, and JPS Health Network EHRs). Consistent with Commission on Cancer requirements, complete information about vital status was available for over 90% of patients through 5 years of follow-up. Covariates of interest included baseline sociodemographic characteristics (age, sex, race/ethnicity, and marital status), health behaviors and status (smoking, alcohol use, and comorbidities), and tumor characteristics (American Joint Committee on Cancer stage, histologic grade, hormone receptor status [for breast cancer], and cancer subtype [for lung cancer]).

### Data Analysis

We used a counterfactual framework^25^ to estimate the average treatment effect on the treated of implementing a DST on survival in the study population. We used flexible parametric models^26,27^ with DST implementation as a time-dependent covariate to estimate standardized 3-year restricted mean survival time (RMST^28-31^) difference and all-cause mortality risk ratio (RR) with 95% confidence limits (CLs) by cancer type, where individuals were eligible for follow-up from the time of diagnosis to death, loss to follow-up, or end of study period (December 31, 2019), whichever occurred first. Estimates were standardized on baseline prognostic factors related to survival for each cancer type including age as a restricted cubic spline with four knots (at 5%, 35%, 65%, and 95%),^32^ race/ethnicity (non-Hispanic Black, Hispanic, non-Hispanic other, and non-Hispanic White), insurance status (uninsured, uninsured enrolled in medical assistance program, Medicaid/Medicare, and other), marital status (unmarried, divorced/separated/widowed, and married), comorbidities (National Cancer Institute comorbidity index^33^ >0 *v* 0), BMI (standard categories of underweight, normal weight, overweight, obese), alcohol use (current or former use *v* never use), tobacco use (current, former, and never), stage (I, II, III, and IV), grade (poorly differentiated or undifferentiated, moderately differentiated, and well differentiated), estrogen receptor status (yes *v* no), and human epidermal growth factor receptor 2 status (yes *v* no) for breast cancer; and age, sex, race/ethnicity, insurance status, marital status, comorbidities, BMI, alcohol use, tobacco use, and stage for colorectal and lung cancers. Some categories were collapsed to reduce the potential for sparse data bias^34^ (eg, few people reported former alcohol use and were combined with current use). In addition, grade was not included in standardization for colorectal and lung cancers because of excessive missing values.

### Adherence With Tool-Based Treatment Recommendations

We recognized that survival effects may depend on the magnitude of adherence with tool-based treatment recommendations before and after implementation. Consequently, we estimated adherence proportions before and after implementation of the DST for additional insight. The DST generates reports of adherence with treatment recommendation for each patient, where adherence is defined as concordance between first-line pathway recommendation and actual treatment initiated, which allowed us to estimate the adherence proportion. Patients who did not initiate treatment (eg, died or lost to follow-up) were excluded when estimating the adherence proportion.

Adherence with tool-based treatment recommendations was unknown before use of the DST, but information from patients in the historical comparison group could be manually entered in the DST to generate a recommendation. An assessment of adherence for all patients in the historical comparison group was infeasible, given resource limitations. Therefore, we randomly sampled 5% of the historical comparison group for breast, colorectal, and lung cancers to determine whether the treatment initiated adhered with the recommendation. One clinical investigator (K.N.), who arrived at the institution after DST implementation, was provided baseline tumor characteristics for the substudy sample to enter in the DST while being blinded to actual treatment received and data that would not have been available at the time of treatment decision making (ie, no post-baseline information) to reduce potential bias in assessing adherence. Treatment recommendations were thus generated for the study subsample, and we estimated the adherence proportion for the historical comparison group.

## RESULTS

Our study population comprised 1,059 patients diagnosed with first primary invasive female breast (n = 323), colorectal (n = 318), or lung (n = 418) cancer. Table 1 summarizes the distributions of sociodemographic, behavioral, and disease characteristics of our study population by intervention (with DST) and the historical comparison (without DST) group. Briefly, the median age of patients with breast cancer was 54 years (IQR, 48-63) at diagnosis and were predominantly racial/ethnic minorities (67%). A substantial proportion of breast cancer patients were uninsured or subsidized care through a hospital-based medical assistance program for uninsured individuals (49%), and 11% were diagnosed with stage IV disease. The median age of patients with colorectal cancer was 56 years (IQR, 51-62), were predominantly male (56%), and predominantly racial/ethnic minorities (66%). Most patients with colorectal cancer were uninsured or subsidized care through a hospital-based medical assistance program for uninsured individuals (69%), and 33% were diagnosed with stage IV disease. The median age of patients with lung cancer was 60 years (IQR, 55-65), were predominantly male (55%), and predominantly non-Hispanic White (55%). A substantial proportion of patients with lung cancer were uninsured or subsidized care through a hospital-based medical assistance program for uninsured individuals (54%), and the majority were diagnosed with stage IV disease (57%).

**TABLE 1.**
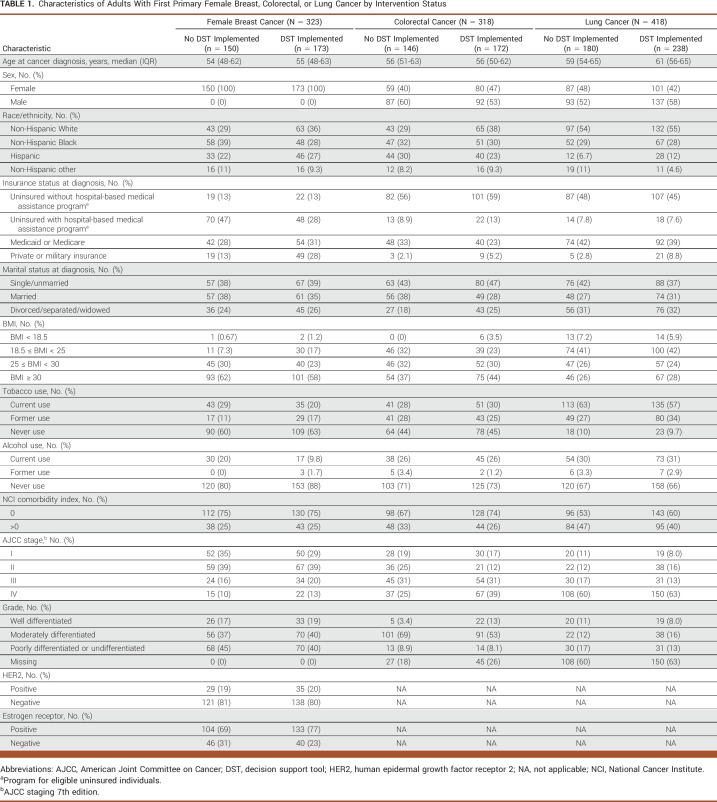
Characteristics of Adults With First Primary Female Breast, Colorectal, or Lung Cancer by Intervention Status

**FIG 1.**
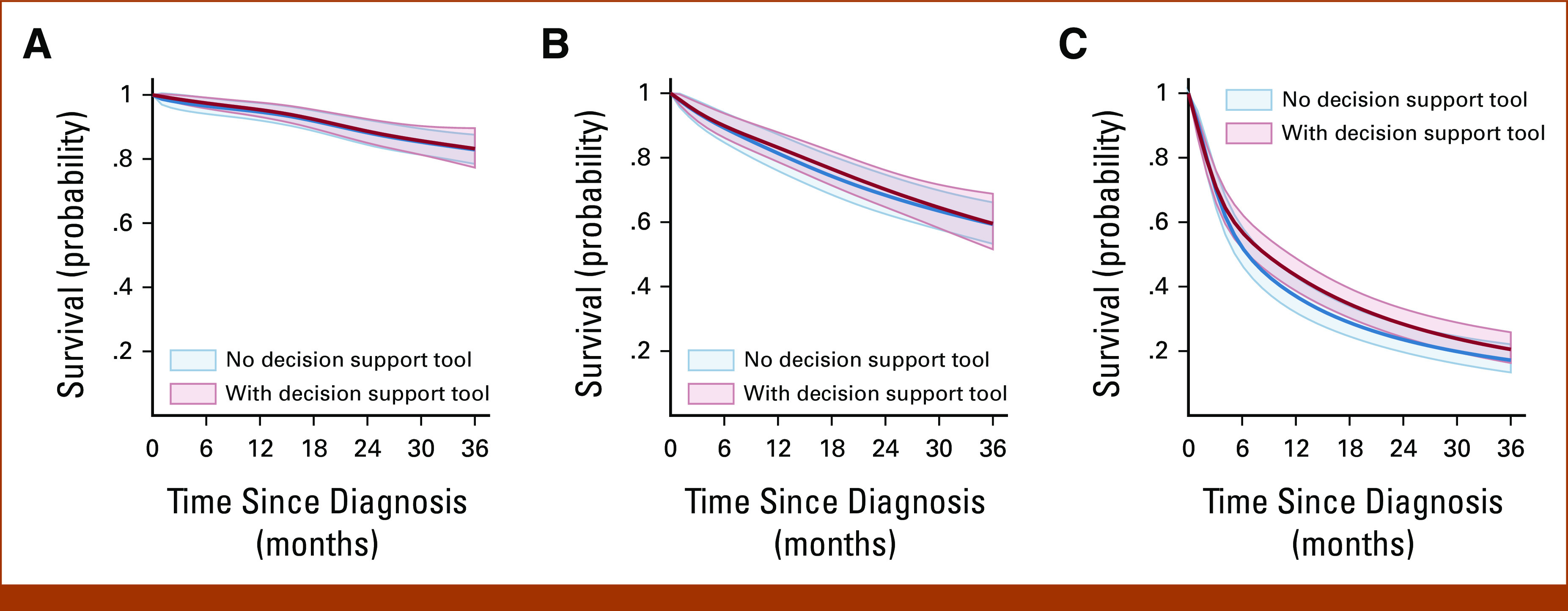
Standardized survival curves for implementation versus no implementation of a decision support tool for cancer treatment among adults diagnosed with a first primary (A) female breast, (B) colorectal, or (C) lung cancer.

Figure 1 illustrates 3-year adjusted survival curves for implementation and no implementation of the DST for breast, colorectal, and lung cancer. Appendix Table A1 summarizes unadjusted results and Table 2 summarizes the estimates and differences in 3-year RMST and 3-year all-cause mortality risk for the three cancer types. The 3-year RMST for female breast cancer was 33 months for implementation (95% CL, 32 to 34) and no implementation (95% CL, 32 to 34) of the DST (RMST difference, –0.16 months, 95% CL, –1.6 to 1.2). The 3-year all-cause mortality risk for breast cancer was 17% for implementation (95% CL, 12 to 24) and no implementation (95% CL, 13 to 22) of the DST (RR, 0.98; 95% CL, 0.62 to 1.6). The 3-year RMST for colorectal cancer was 28 months (95% CL, 26 to 29) for implementation and 27 months (95% CL, 26 to 29) for no implementation of the DST (RMST difference, 0.64 months; 95% CL, –1.7 to 3.0). The 3-year all-cause mortality risk for colorectal cancer was 41% for implementation (95% CL, 33 to 51) and no implementation (95% CL, 35 to 48) of the DST (RR, 1.0, 95% CL, 0.78 to 1.3). The 3-year RMST for lung cancer was 15 months (95% CL, 13 to 16) for implementation and 13 months (95% CL, 12 to 14) for no implementation of the DST (RMST difference, 1.7 months; 95% CL, –0.26 to 3.7). The 3-year all-cause mortality risk for lung cancer was 79% (95% CL, 75 to 84) for implementation and 83% (95% CL, 79 to 87) for no implementation of the DST (RR, 0.95; 95% CL, 0.88 to 1.0).

**TABLE 2.**
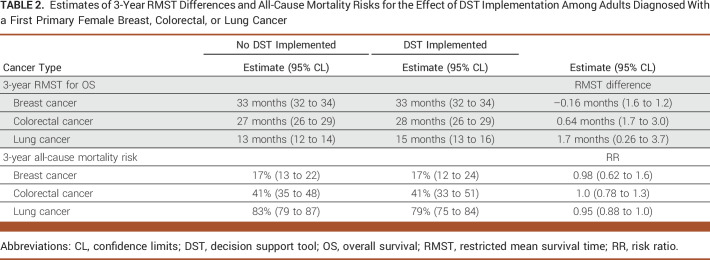
Estimates of 3-Year RMST Differences and All-Cause Mortality Risks for the Effect of DST Implementation Among Adults Diagnosed With a First Primary Female Breast, Colorectal, or Lung Cancer

Adherence with tool-based treatment recommendations in the historical comparison group was 72% for breast cancer, 76% for colorectal cancer, and 75% for lung cancer. Adherence with tool-based treatment recommendations in the intervention group was 91% for breast (19% increase), 95% for colorectal (19% increase), and 91% for lung (16% increase).

## DISCUSSION

Our results suggest that implementation of a DST has nominal effect on 3-year survival for patients with breast cancer. Implementation of a DST also has nominal effect on 3-year survival for patients with colorectal cancer, but given imprecision, the 95% CL suggests that 1.7 month shorter survival or 3.0 month longer survival is compatible with our data. The largest effect of implementing the DST was observed for lung cancer with 1.7 month longer survival over a 3-year follow-up period, but up to 3.7 month longer survival (or 12% lower relative mortality risk) is compatible with these data. These nominal differences were observed despite adherence with tool-based treatment recommendations exceeding 90% after implementation (16%-19% increases) across cancer types from the period before tool implementation. Nevertheless, interpretation of our findings requires several considerations.

Inference from any study assumes exchangeability between comparison groups, which is an untestable assumption. Violations of exchangeability manifest as confounding or selection biases. For example, violations of exchangeability in single-arm trials with historical comparison can arise from temporal drift of prognostic characteristics or concurrent interventions that affect the outcome (ie, confounding bias). Exchangeability may be violated if unmeasured factors of poor prognosis were distributed differently between the intervention (ie, DST implementation) and historical comparison (ie, usual care on the basis of provider discretion) groups. The prevalence of poor prognostic factors would have to be higher in the intervention group to explain the observed nominal effect on survival. Nevertheless, other than precision, we observed negligible differences between crude and adjusted estimates despite adjusting for numerous baseline covariates. For example, the 95% CL for 3-year mortality ratios were similar between crude and adjusted analysis for breast cancer (crude, 0.59 to 1.9; adjusted, 0.62 to 1.6). Consequently, confounding bias related to different distributions of baseline prognostic factors may not explain our findings.

Exchangeability may also be violated because of changes in treatment over time (ie, postbaseline interventions), whether from changes in standard of care or participation in clinical trials. A switch to new and more effective treatments would be a greater concern in the intervention group. If new treatments increased survival in the intervention group, then our results may overestimate the effect of implementing a DST on survival (ie, the true effect of a DST could be smaller), which may be most consequential for lung cancer. Nevertheless, our eligibility period was brief and occurred before widespread use of novel first-line immunotherapy or targeted therapy for cancers of interest (Appendix Table A2). In addition, participation in oncology clinical trials for our population was rare during the study period. Consequently, we speculate modest effect of confounding bias related to changing treatments over time.

Despite longer follow-up in our study than most prior studies, follow-up duration is a consideration when interpreting our estimates. The main value of extended follow-up is a larger number of events, which enhances the precision of effect estimates. One measure of precision is the CL ratio,^35^ where the numerator is the upper 95% CL and the denominator is the lower 95% CL. CL ratios closer to one for relative measures, such as RRs, suggest more precise estimates. The CL ratio is 2.6 for breast cancer, 1.7 for colorectal cancer, and 1.1 for lung cancer in our study. These measures weaken the idea that additional follow-up would provide substantial gains in precision for lung and colorectal cancers, but additional follow-up may provide insight for breast cancer. Nevertheless, the results for lung cancer provide some insight about potential effects with longer follow-up, given rapid mortality in this population. If the effect observed for lung cancer translates to breast and colorectal cancers, then longer follow-up may not necessarily result in dramatic improvements in survival after implementing a DST because the modest separation of survival curves occurred early in the follow-up period. We also deliberated including surrogate outcomes, such as progression-free survival, to increase the number of events and enhance precision, but progression-free survival is often a poor surrogate for OS.^36-38^ For example, unlike trials, disease progression is not systematically assessed in practice. Lack of systematic assessment contributes to misclassification of progression and measurement errors in time to progression.

Our results may not be generalizable outside of safety-net health systems. For example, our population has higher prevalence of adverse prognostic characteristics and adverse social determinants of health that may override potential benefit of implementing a DST.^20^ This phenomenon may not occur in populations outside of safety-net health systems. In addition, DSTs have been developed by commercial vendors, payers, and health systems using different criteria for treatment recommendations.^39^ Clinical practice guidelines are commonly used as a foundation for treatment recommendations embedded within DSTs, but tool developers use different processes and criteria to prioritize recommendations and update recommendations when new evidence emerges.^39^ For example, treatment recommendations may vary across tools depending on prioritization of effectiveness, toxicity, and cost during development, which may lead to more or less restrictive recommendations. Despite general recommendations for developing clinical pathways embedded in DSTs^4^ and criteria for comparing the development, implementation, and analytics of these pathways,^3^ no standardized criteria currently exist for guiding treatment recommendations within DSTs. Consequently, the effects on clinical outcomes may vary depending on the selected tool.^40^

Several studies evaluated the effect of DSTs on outcomes such as adherence with tool-based treatment recommendations and cost,^6,7^ but few studies evaluated effects on survival. We identified three studies^12-14^ conducted in the United States that reported effects on survival. Table 3 summarizes characteristics of these studies and the current study. Two studies compared survival between patients concordant and discordant with treatment recommendations,^12,13^ but this comparison is problematic. Comparisons of concordant and discordant groups provide little insight about the effect of DST implementation because both groups arose after the tool was implemented. In addition, patients that were discordant with treatment recommendations have contraindications (eg, comorbidities, frailty, etc) or personal objections to treatment recommendations. Patients with contraindications are thus ineligible for treatment, which violates the positivity assumption (ie, nonpositivity) and undermines inference. The positivity assumption requires that groups being compared are eligible for the exposure or intervention of interest within covariate strata.^41,42^ Positivity is guaranteed in a randomized trial because only individuals eligible for the intervention group or comparison group are included in the study population, but observational designs require additional considerations to avoid positivity violations. Nonpositivity is distinct from confounding bias, and covariate adjustment cannot resolve nonpositivity.^41,42^ The consequence of nonpositivity is that the effect estimate comparing groups has no practical interpretation.

**TABLE 3.**
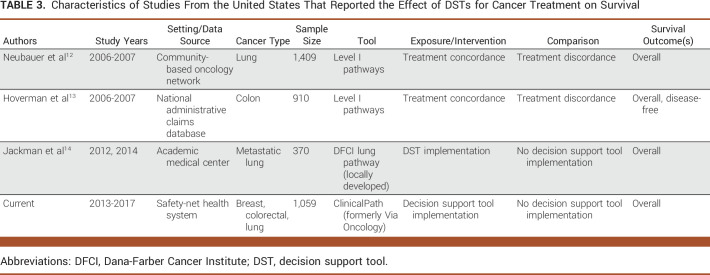
Characteristics of Studies From the United States That Reported the Effect of DSTs for Cancer Treatment on Survival

Given challenges with positivity and unless a randomized trial of DST implementation is conducted, a quasi-experimental design that emulates a single-arm trial with historical comparison may be a pragmatic alternative for generating evidence. Jackman et al^14^ also used this design and reported similar median survival before and after implementation of a DST (11.2 months and 10.7 months, respectively). Nevertheless, this study was limited to patients with stage IV non–small-cell lung cancer and included only 1 year of follow-up. The results from the study by Jackman et al^14^ are also difficult to compare with our study because no effect estimate was reported,^14^ and the reported median survival is inconsistent with the standard definition. Median survival is defined as the time at which survival probability in the population is 50% (ie, half the study population remains alive), but this definition was not met in the study by Jackman et al.^14^ Median survival was also inestimable in our breast and colon cancer populations, which was one reason we reported RMST.^28-31^

DSTs for cancer treatment arguably have benefits that may justify implementation such as improvements in process outcomes such as care standardization, increased use of evidence-based treatments, and efficiency.^6,7^ Our results may be useful for raising awareness that improved process outcomes may not translate to improved patient outcomes in some care delivery settings. One explanation is a potential threshold effect, where improvements in survival may not be observed if adherence with treatment recommendations reaches a sufficiently high proportion within a population that additional increases in adherence with recommendations will have little effect on survival. This threshold effect could occur in the period before or after DST implementation and may depend on the effectiveness of treatments recommended by the tool (ie, the underlying evidence). For example, recent systematic reviews of US Food and Drug Administration approvals of cancer drugs between 2000 and 2016 estimate that median survival improved by only 2.4 months for drugs approved between 2000 and 2016,^43^ and 2.8 months for drugs approved between 2003 and 2021.^44^ Consequently, even 100% adherence with treatment recommendations for a population may have small effect on survival. Greater improvement in population survival after DST implementation is possible with smaller increases in adherence to treatment recommendations if future treatments have larger effects on survival and are used to treat a sufficiently large proportion of the population. Given the rapidly changing landscape of cancer treatment recommendations and concerns about DSTs, including lack of transparency in treatment recommendations, cost of implementation and maintenance, and adverse effects on patient-provider relationship,^3^ continuous evaluation of DSTs is needed to understand the context in which these tools can improve patient outcomes.

## Figures and Tables

**TABLE A1.**
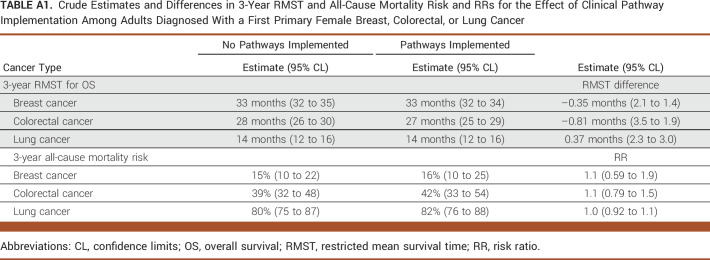
Crude Estimates and Differences in 3-Year RMST and All-Cause Mortality Risk and RRs for the Effect of Clinical Pathway Implementation Among Adults Diagnosed With a First Primary Female Breast, Colorectal, or Lung Cancer

**TABLE A2.**
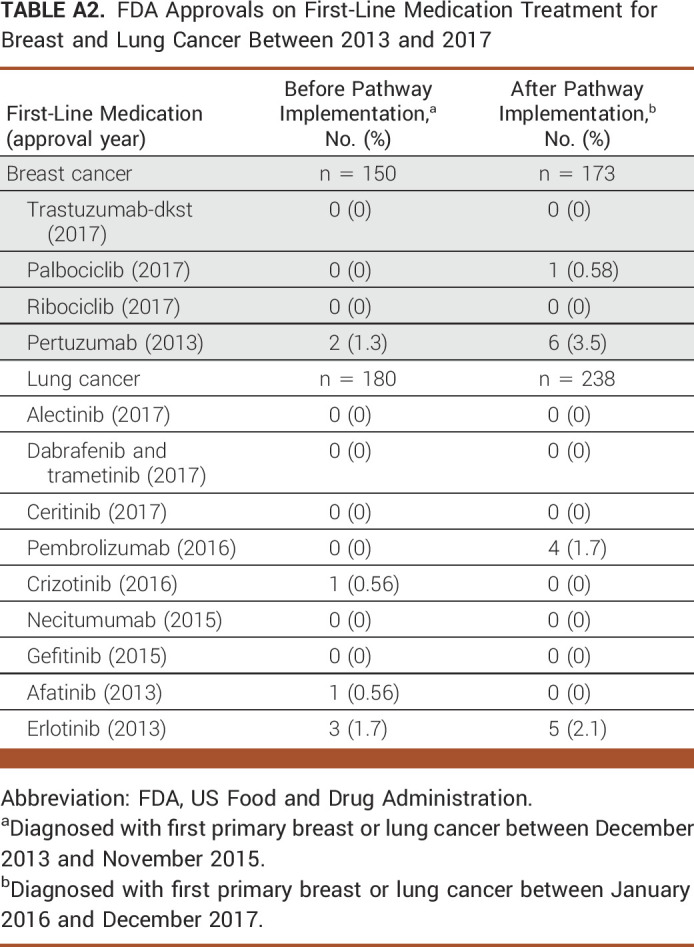
FDA Approvals on First-Line Medication Treatment for Breast and Lung Cancer Between 2013 and 2017

## Data Availability

The data that support the findings of this study are available on reasonable request to the corresponding author and review by the JPS Health Network External Data Governance Committee (research@jpshealth.org).
